# Evolution and Stress Responses of *Gossypium hirsutum SWEET* Genes

**DOI:** 10.3390/ijms19030769

**Published:** 2018-03-08

**Authors:** Wei Li, Zhongying Ren, Zhenyu Wang, Kuan Sun, Xiaoyu Pei, Yangai Liu, Kunlun He, Fei Zhang, Chengxiang Song, Xiaojian Zhou, Wensheng Zhang, Xiongfeng Ma, Daigang Yang

**Affiliations:** State Key Laboratory of Cotton Biology, Institute of Cotton Research of Chinese Academy of Agricultural Sciences, Anyang 455000, China; liwei@caas.cn (W.L.); renzhongying123@gmail.com (Z.R.); wangzhenyu01@caas.cn (Z.W.); sunkuan@caas.cn (K.S.); peixiaoyu@caas.cn (X.P.); liuyangai@caas.cn (Y.L.); hekunlun@caas.cn (K.H.); zhangfei@caas.cn (F.Z.); songcx526@gmail.com (C.S.); zhouxiaojian@caas.cn (X.Z.); zhangwensheng@caas.cn (W.Z.)

**Keywords:** cotton, SWEET, evolution, stress response, artificial selection

## Abstract

The SWEET (sugars will eventually be exported transporters) proteins are sugar efflux transporters containing the MtN3_saliva domain, which affects plant development as well as responses to biotic and abiotic stresses. These proteins have not been functionally characterized in the tetraploid cotton, *Gossypium hirsutum*, which is a widely cultivated cotton species. In this study, we comprehensively analyzed the cotton *SWEET* gene family. A total of 55 putative *G. hirsutum* *SWEET* genes were identified. The *GhSWEET* genes were classified into four clades based on a phylogenetic analysis and on the examination of gene structural features. Moreover, chromosomal localization and an analysis of homologous genes in *Gossypium arboreum*, *Gossypium raimondii*, and *G. hirsutum* suggested that a whole-genome duplication, several tandem duplications, and a polyploidy event contributed to the expansion of the cotton *SWEET* gene family, especially in Clade III and IV. Analyses of *cis*-acting regulatory elements in the promoter regions, expression profiles, and artificial selection revealed that the *GhSWEET* genes were likely involved in cotton developmental processes and responses to diverse stresses. These findings may clarify the evolution of *G. hirsutum SWEET* gene family and may provide a foundation for future functional studies of SWEET proteins regarding cotton development and responses to abiotic stresses.

## 1. Introduction

In plants, sugars are an essential carbon and energy source for growth and development. Additionally, unicellular organisms use sugars as a principal carbon source for energy production and as nutrients supplements. Multicellular organisms acquire sugars, such as glucose or sucrose, which are transported from source to sink organs and are associated with the cellular exchange of carbon and energy [1,2]. Sugar transport is controlled by the SUT (sucrose transporter), SWEET (sugars will eventually be exported transporters; sugar effluxer) proteins and so on [3,4,5]. The SUT proteins have been detected in many species and are responsible for the loading sucrose into phloem across cell membranes [6]. Meanwhile, the SWEET proteins were identified as sugar effluxers that transport monosaccharides or disaccharides across intracellular or plasma membranes [7,8]. The SWEET proteins are characterized by seven conserved MtN3_saliva transmembrane domains, which comprise a pair of PQ-loop repeats. Members of the *SWEET* family have been identified in plants, human, protozoa, metazoa, fungi, bacteria, and archaea [9,10,11]. Recent investigations detected 17 and 21 *SWEET* genes in *Arabidopsis thaliana* and rice, respectively, and up to 52 in soybean [12,13,14]. A phylogenetic analysis revealed that these genes can be classified into four clades [12,14,15]. Clade members interact with each other to facilitate the efficient diffusion of sugars for required cellular activities, but are also critical for nectar production and seed, embryo, and pollen development [2,12,16,17]. For example, *AtSWEET2*, *AtSWEET16* and *AtSWEET17* not just collectively mediate vacuolar sugar transport, and that exhibit strong expression in root cortical cells [16,17,18].

The *SWEET* gene family has been investigated in *A. thaliana* [12], rice [13], grapevine [19], soybean [14], tomato [20], potato [15], cucumber [21] and wheat [22]. The first identified plant *SWEET* gene is *AtSWEET1*, which encodes a protein involved in supplying nutrients to the gametophyte or nectary [12]. Moreover, *AtSWEET8* is required for pollen viability [12], while *AtSWEET11* and *AtSWEET12* are localized in the plasma membrane, where they function as the predominant regulators of sucrose, glucose and fructose fluxes and contribute to the response to cold stress and water deficit conditions [3,23]. The overexpression of *AtSWEET16* results in enhanced freezing tolerance [24]. In rice, *OsSWEET1a*, *OsSWEET2a*, *OsSWEET3a*, *OsSWEET4*, *OsSWEET5*, and *OsSWEET15* are highly expressed in flowers or panicles at different developmental stages [13]. An earlier study confirmed that *OsSWEET14-*silenced mutants produced relatively small seeds and exhibited delayed growth [13]. Additionally, the expression levels of most *SlSWEET* genes were observed to be upregulated several fold in response to salt and temperature stresses in tomato [20]. Furthermore, *BnSWEET12* expression is positively induced by abscisic acid (ABA), gibberellin (GA) and brassinosteroid (BR) treatments in oilseed rape [25]. Therefore, *SWEET* genes are involved in diverse physiological and biochemical pathways [15,19,26].

Cotton is one of the most important fiber crops worldwide, and cottonseed oil can be used as biodiesel [27,28]. *Gossypium hirsutum*, a tetraploid cotton, is the most widely cultivated cotton species, that supplies about 98% of the produced textile fibers [29]. However, to date, there have been relatively few studies conducted on cotton SWEET proteins. A recent study confirmed that the expression of a certain *SWEET* gene in cotton is induced by a transcription activator-like effector, which influences *Xanthomonas citri* subsp. *malvacearum* pathogenicity [30]. The suppressed expression of *GhSCP2D*, which encodes a putative sterol carrier protein, activates a cohort of *SWEET* genes, resulting in the elongation of cotton fiber cells [31]. Thus, a comprehensive analysis of the *G. hirsutum SWEET* gene family is essential. The completion of the tetraploid upland cotton genome sequencing project has enabled a genome-wide identification and functional analysis of the *SWEET* gene family in cotton.

In the present study, we conducted a genome-wide analysis to identify 55 putative *SWEET* gene family members in *G. hirsutum*. We also analyzed their phylogenetic relationships, structures, localization, genetic variations, and expression profiles in different tissues and in response to cold, heat, salt, and drought stresses. Our results provide the basis for future investigations of the roles of the SWEET proteins in developing cotton plants.

## 2. Results

### 2.1. Identification and Characterization of the G. hirsutum SWEET Gene Family

A total of 58 putative *G. hirsutum SWEET* genes were detected, and their coding sequences were manually confirmed using the FGENESH gene prediction tool as well as cDNA sequences after performing PCR with gene-specific primers. Consequently, three genes whose genome sequences and coding sequences were very short were excluded, and the coding sequences of 22 *SWEET* genes were modified from their initial annotation in the CottonGen database. Finally, the 55 *SWEET* genes confirmed in the *G. hirsutum* genome were named *GhSWEET1*–*GhSWEET55* according to their chromosomal position (Tables 1 and S2). The number of amino acids, Molecular weight (MW) and Isoelectric point (pI) were calculated on the basis of the predicted protein sequences. Furthermore, for comparative analyses, 31 and 30 *SWEET* genes were identified in the *Gossypium raimondii* and *Gossypium arboreum* genomes, respectively, using the same methods (Table S3).

### 2.2. Phylogenetic Analysis and Structural Features of the SWEET Gene Family

**Figure 1:**
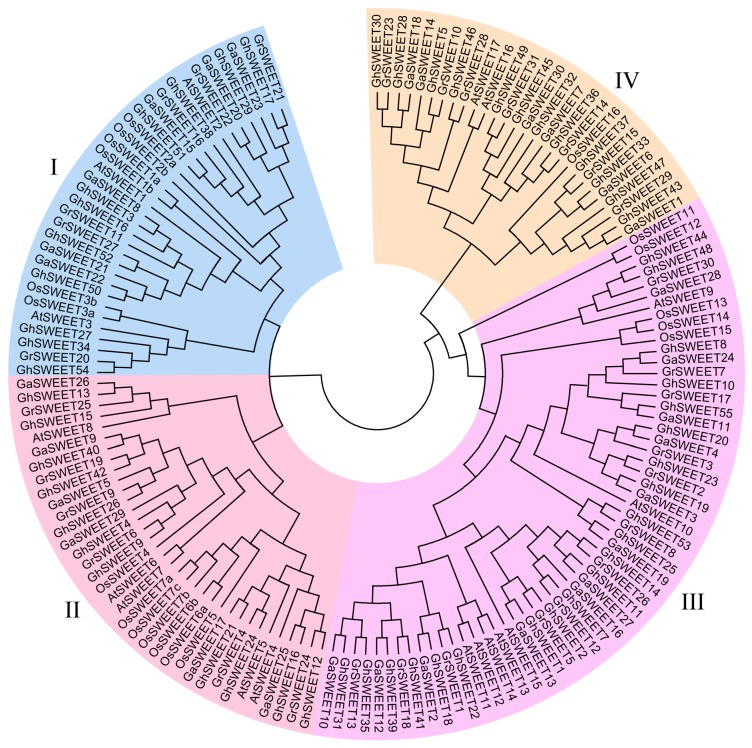
Neighbor-joining phylogenetic tree of *SWEET* genes from three cotton species, *A. thaliana*, and rice. The *SWEET* genes were classified into four clades, which are indicated by different colors.

To investigate the evolutionary relationships among the *SWEET* genes from three *Gossypium* species, *A. thaliana*, and rice, an unrooted phylogenetic tree was constructed using the neighbor-joining method (Figure 1). The *SWEET* genes were clearly divided into four classes (Clades I, II, III, and IV), consistently with the reported results for *A. thaliana* and rice *SWEET* genes [12,14]. The largest clade was Clade III, which consisted of five *OsSWEET* genes, seven *AtSWEET* genes, and 45 cotton *SWEET* genes (12 *G. raimondii*, 12 *G*. *arboreum*, and 21 *G*. *hirsutum SWEET* genes). Clade I comprised six *OsSWEET* and three *AtSWEET* genes, while Clade II included seven *OsSWEET* and five *AtSWEET* genes. Clades I and II contained an equal number of cotton *SWEET* genes, with six *G. raimondii*, six *G*. *arboreum*, and eleven *G*. *hirsutum SWEET* genes. Clade IV consisted of only two *A. thaliana* and one rice *SWEET* genes, but seven *G. raimondii*, six *G*. *arboreum*, and 12 *G*. *hirsutum SWEET* genes. These results suggested that *SWEET* gene families expanded, especially in Clade III and IV, during cotton evolution.

**Figure 2:**
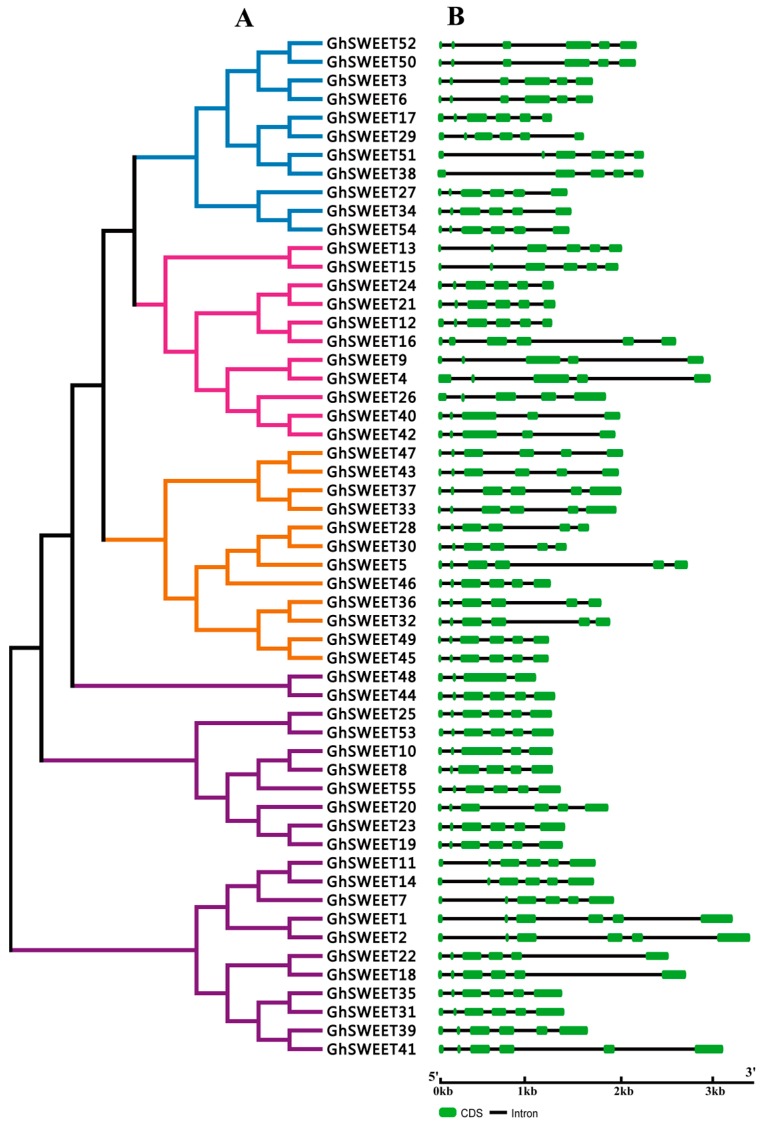
Phylogenetic relationships and structural features of *GhSWEET* genes. (**A**) Neighbor-joining phylogenetic tree of *GhSWEET* genes. The *SWEET* genes were classified into four clades, and blue, pink, purple, and orange represent Clades I, II, III, and IV, respectively. (**B**) Exon-intron organization of *GhSWEET* genes. Green and black correspond to exons and introns, respectively.

To elucidate the structural features of *G. hirsutum SWEET* genes, the exon-intron organization was analyzed. Gene structures generally exhibited a highly conserved distribution of exons and introns within the same clade or subclade (Figure 2B). Specifically, most Clade I genes (except *GhSWEET38*) and all Clade IV genes contained five introns. Meanwhile, Clade II was divided into two subclades, IIa and IIb. The six subclade IIa genes had five introns, while the subclade IIb genes contained four introns. Of the 21 Clade III genes, 19 had five introns, while *GhSWEET10* comprised four introns and *GhSWEET48*, which was relatively short, contained three introns. These results revealed that introns were distributed consistently among members of the same phylogenetic clade.

### 2.3. Chromosomal Distribution and Homology of G. hirsutum SWEET Genes

**Figure 3:**
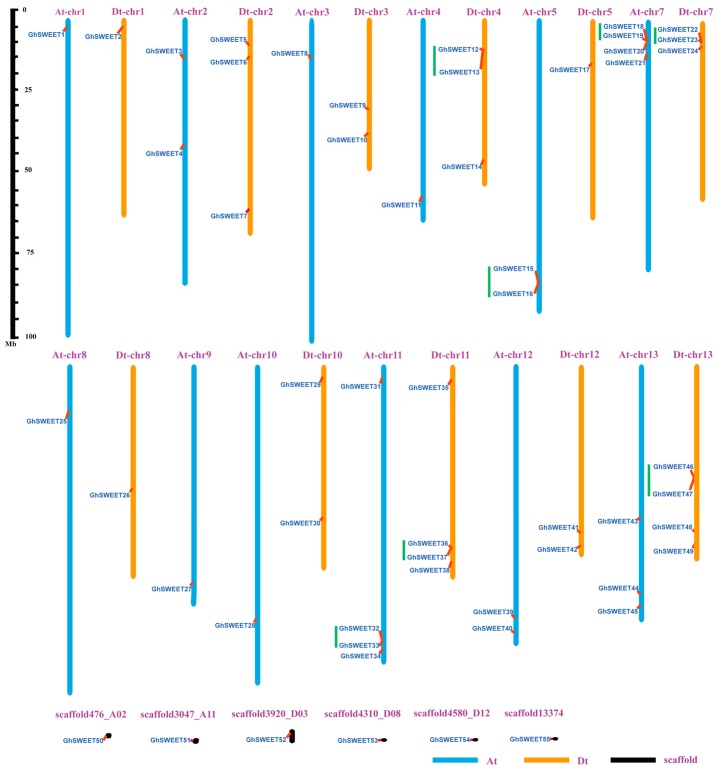
Chromosomal distribution of *GhSWEET* genes. Blue, orange and black indicate the At subgenome chromosomes, Dt subgeome chromosomes, and scaffolds, respectively. Green lines indicate tandem duplicated genes. The scale is provided in megabases.

*G. hirsutum* is a model for polyploid crop domestication, whose complex allotetraploid genome (AtDt, where “t” stands for tetraploid) was sequenced and oriented to 26 pseudochromosomes. Among the 55 *G. hirsutum SWEET* genes, 49 were localized on 12 of 13 At chromosomes and 11 of 13 Dt chromosomes (Figure 3). Six genes (*GhSWEET50*, *GhSWEET51*, *GhSWEET52*, *GhSWEET53*, *GhSWEET54*, and *GhSWEET55*) were distributed on scaffolds whose exact locations on chromosomes were not determined. Chromosomes At-chr7, At-chr11, Dt-chr11 and Dt-chr13 contained four genes, while chromosomes Dt-chr2, Dt-chr4, Dt-chr7, and Dt-chr3 included three genes each. Interestingly, 14 genes were clustered in seven tandem duplication regions at the chromosomes Dt-chr4, At-chr5, At-chr7, Dt-chr7, At-chr11, Dt-chr11, and Dt-chr13. Of these, similar clusters were identified between homoeologous chromosomes (At-chr7 and Dt-chr7 as well as At-chr11 and Dt-chr11).

**Figure 4:**
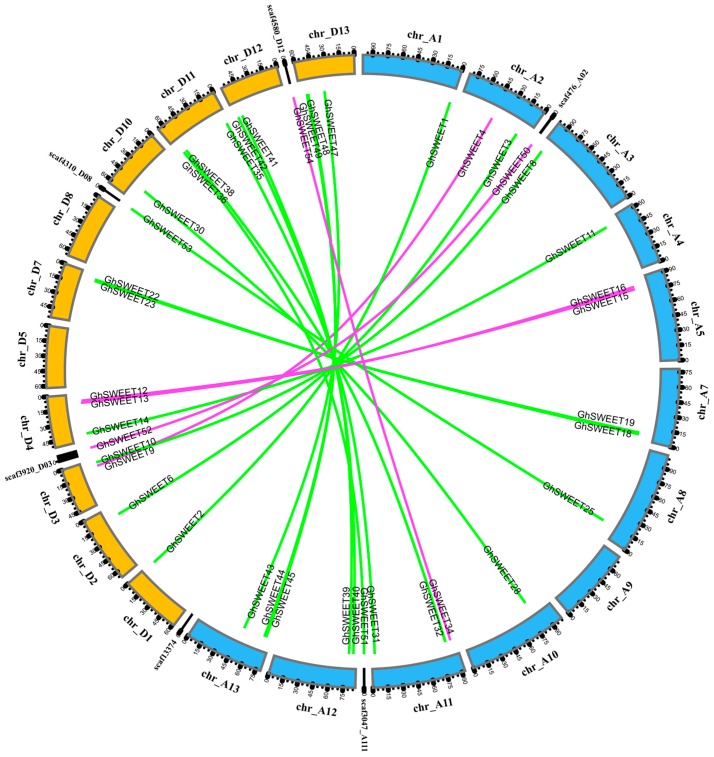
Distribution of paralogous *SWEET* gene pairs between *G. hirsutum* At and Dt subgenomes. The 12 At subgenome chromosomes, 11 Dt subgenome chromosomes, and six scaffolds are indicated by different colors, with their names on the periphery. Green lines link the homologous genes located on homoeologous chromosomes in the At and Dt subgenomes. Purple lines link the homologous genes located on non-homoeologous chromosomes in the At and Dt subgenomes and scaffolds.

Most angiosperms have undergone at least one paleopolyploidy event during evolution [32,33]. The genome of *G. hirsutum* (AtDt), which is a typical allotetraploid, underwent a diploidization following the divergence of this cotton species from its diploid ancestors, *G. arboreum* (AA) and *G. raimondii* (DD)*.* Firstly, we analyzed the *SWEET* paralogs between the At and Dt subgenomes (Figure 4). A total of 23 paralogous gene pairs were identified, with 18 of them anchored across eight homoeologous chromosomes pairs. To further clarify the divergence during cotton evolution, we analyzed the orthologous *SWEET* genes between the *G. hirsutum* Dt subgenome and the corresponding ancestral D diploid genome (*G. raimondii*), as well as between the *G. hirsutum* At subgenome and the corresponding ancestral A diploid genome (*G. arboreum*) (Figure S1). A total of 28 genes in the Dt subgenome had orthologs in the *G. raimondii* genome, while 23 genes in the At subgenome had orthologs in the *G. arboreum* genome. This revealed that the collinear relationship of the *SWEET* genes between the Dt subgenome and the *G. raimondii* genome was higher than that between the At subgenome and the *G. arboreum* genome, indicating either that more At subgenome *SWEET* genes were lost during evolution, or that genome sequencing was incomplete, leading to the identification of only a portion of the existing genes.

### 2.4. Analysis of Cis-Acting Regulatory Elements in the Putative GhSWEET Promoters

Plant *SWEET* genes are associated with responses to abiotic stresses [34,35,36]. Therefore, we analyzed the 1.5-kb sequence upstream of the ATG start codon using the PlantCARE database to detect putative *cis*-acting regulatory elements [37]. The *GhSWEET* promoters contained multiple conserved regulatory elements responsive to phytohormones and environmental stresses (Table S4). Elements related to defense stress, heat, GA, salicylic acid (SA), and drought were widely distributed across the promoter regions of 43, 42, 38, 36, and 35 *SWEET* genes, respectively. According to the regulatory elements in their promoters, 31 *GhSWEET* genes were responsive to methyl jasmonate (MeJA), while 25 genes were responsive to ABA and ethylene. In contrast, low temperatures and auxin appeared to affect a low number of *GhSWEET* genes. These findings implied that *GhSWEET* genes were involved in various regulatory mechanisms in cotton plants’ response to stresses.

### 2.5. G. hirsutum SWEET Gene Expression Profiles in Different Tissues

**Figure 5:**
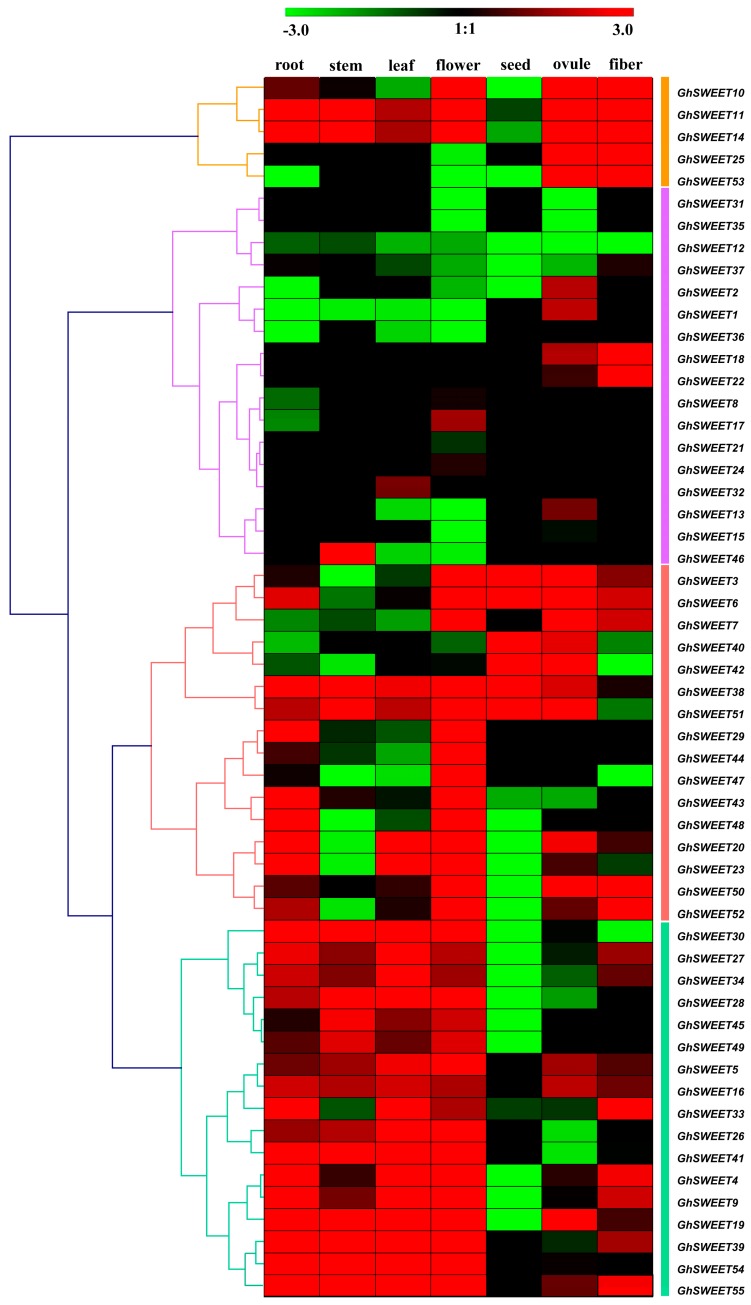
Expression profiles of *GhSWEET* genes in different tissues. *GhSWEET* gene expression profiles are divided into four groups. The FPKM values were calculated by RNA-seq data, and shown as a heat map. The colored bar represents the scale of the relative expression levels.

To investigate the roles of *GhSWEET* genes in different developmental processes, we used publicly available RNA-seq data to analyze *SWEET* gene expression patterns in seven different *G. hirsutum* tissues (root, stem, leaf, flower, ovule, seed, and fiber). The *SWEET* genes were clustered in four groups according to their expression profiles (Figure 5). The five Group I genes were highly expressed in the ovule and fiber. Meanwhile, the Group II genes, *GhSWEET1*, *GhSWEET2*, and *GhSWEET13*, were expressed specifically in the ovule, while most of the Group III genes were expressed in the root and flower. Moreover, the genes in the first clade of Group III (*GhSWEET3*, *GhSWEET6*, *GhSWEET7*, *GhSWEET40*, *GhSWEET42*, *GhSWEET38*, and *GhSWEET51*) were very highly expressed in the seed and ovule. Additionally, the Group IV genes exhibited diverse expression patterns, primarily in six tissues (root, stem, leaf, flower, ovule, and fiber). These results suggested that the *SWEET* genes were important for vegetative and reproductive growth.

### 2.6. Stress-Induced Expression Patterns of G. hirsutum SWEET Genes

**Figure 6:**
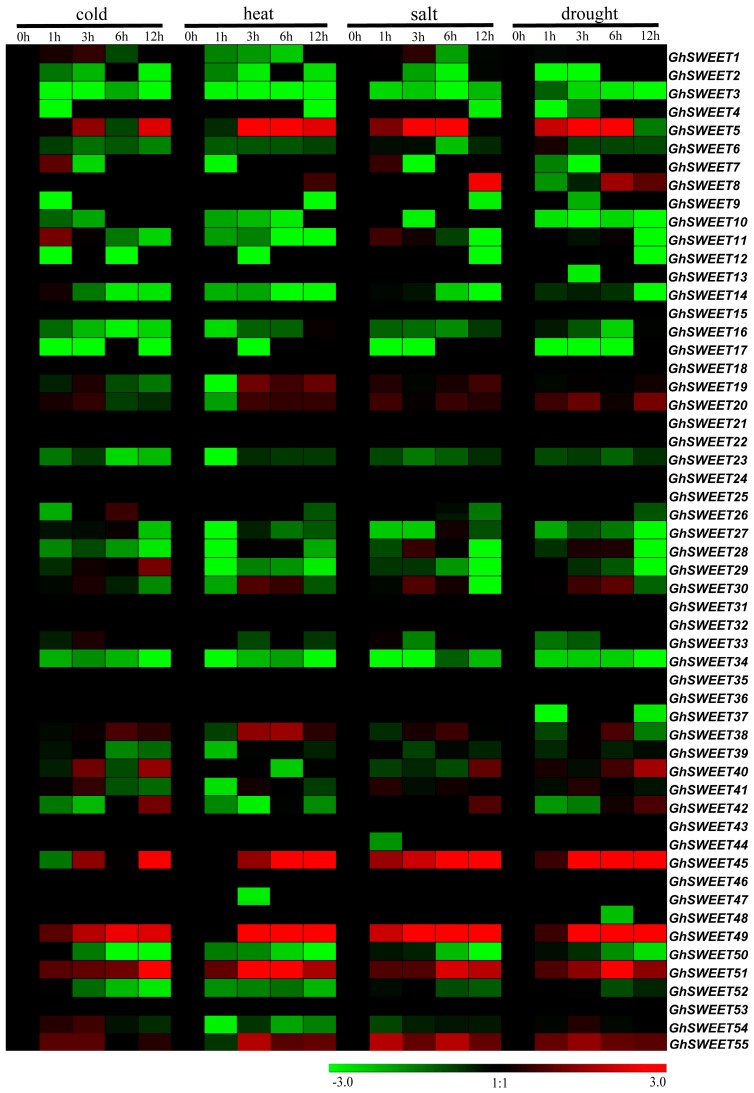
Expression patterns of *GhSWEET* genes in response to various stresses. The ratios of FPKM between treatments (at 1, 3, 6 and 12 h) and controls (at 0 h) were calculated by RNA-seq data, and shown as a heat map. The colored bar represents the scale of the relative expression levels.

Plants are often subjected to multiple abiotic stresses during growth and development. Thus, the expression patterns of the 55 *GhSWEET* genes were analyzed in plants exposed to different durations of cold, heat, salt, and drought stresses for different times by RNA-seq data downloaded from the public database. The expression of some *SWEET* genes was significantly affected by these treatments (Figure 6). The expression levels of six genes (*GhSWEET5*, *GhSWEET20*, *GhSWEET45*, *GhSWEET49*, *GhSWEET51*, and *GhSWEET55*) were increased by all four abiotic stresses. *GhSWEET5*, *GhSWEET49*, and *GhSWEET55* expression levels were upregulated at 6 h but decreased quickly at 12 h under heat and salt stress conditions. The paralogous genes *GhSWEET45* and *GhSWEET49* exhibited similar expression patterns in response to multiple stresses. Overall, cold stress produced lower *SWEET* gene expression levels than the other types of stress. Additionally, some genes were differentially expressed at only one time point of a particular stress treatment. For example, *GhSWEET11* expression was upregulated after 1 h of cold treatment, but was subsequently downregulated.

**Figure 7:**
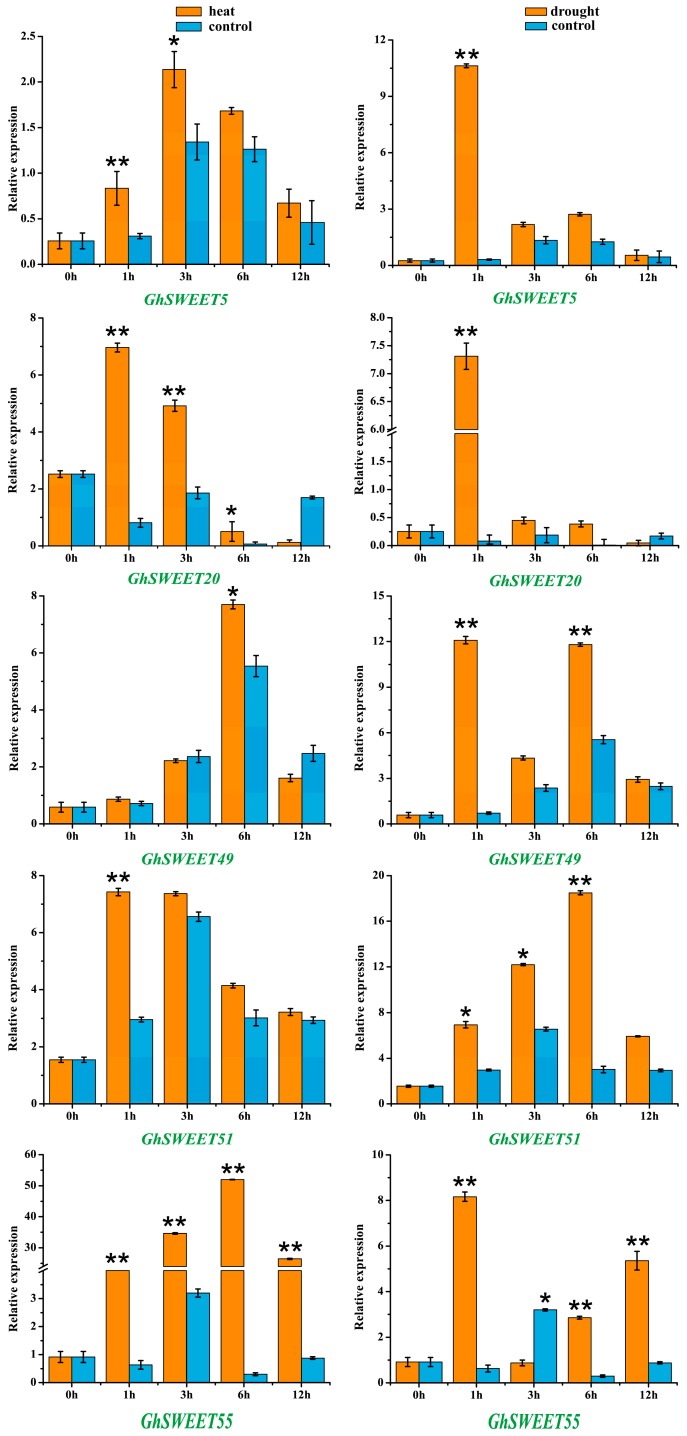
Relative expression levels of the selected *GhSWEET* genes in response to heat and drought stresses. The *GhHis3* was used as an internal control. Relative gene expression levels were calculated based on the 2^−ΔΔ*C*T^ method. The mean expression values were calculated on the basis of three independent replicates. Error bars indicate the standard deviations of three independent experiments. The relative expression levels of selected *GhSWEET* genes after treats were compared with the controls at the same time point. Significant differences were determined by *t*-test (* *p* < 0.05; ** *p* < 0.01).

To further verify *SWEET* gene expression patterns in response to stresses, we selected five *SWEET* genes whose expression was induced by heat and drought conditions, and examined their expression profiles following exposure to 38 °C or 20% PEG 6000 (Figure 7). The qRT-PCR data revealed that the expression levels of the five genes were significantly upregulated by these two treatments. The *GhSWEET20* and *GhSWEET51* transcript levels quickly peaked after 1 h of heat treatment, whereas the increased expression of the *GhSWEET5* transcript occurred after 3 h. Meanwhile, the *GhSWEET49* and *GhSWEET55* expression levels gradually increased, peaking after 6 h. In contrast, the drought-induced transcription of four *SWEET* genes (*GhSWEET5*, *GhSWEET20*, *GhSWEET49* and *GhSWEET55*) peaked after 1 h of stress induction, but then rapidly decreased afterward. These results implied that *GhSWEET* genes might enhance the adaptability of plants to diverse abiotic stresses.

### 2.7. Genetic Variations and Artificial Selection of GhSWEET Genes During Cotton Domestication

**Figure 8:**
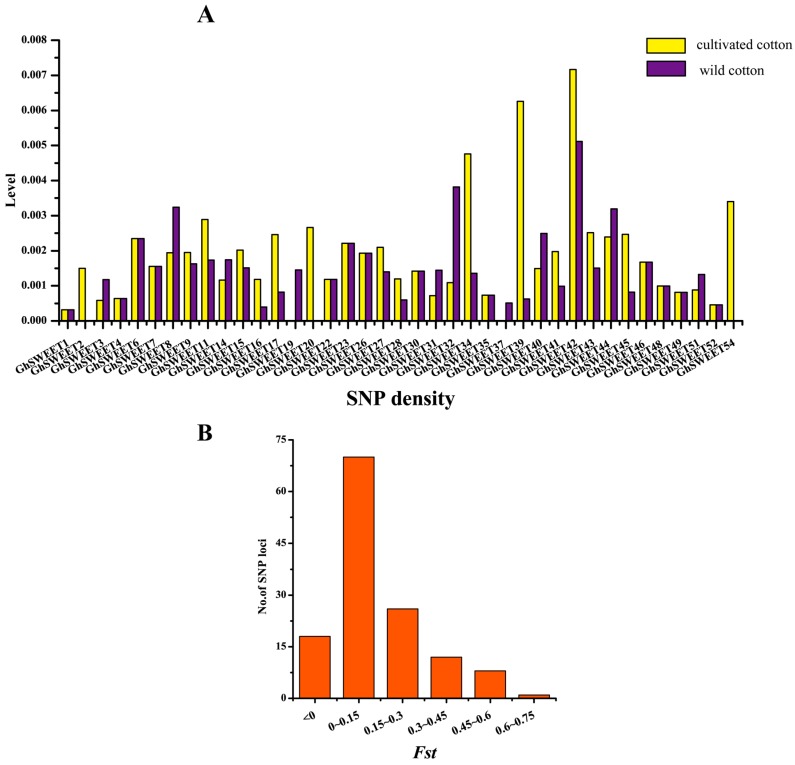
Genetic variations in *G. hirsutum SWEET* genes. (**A**) SNP density in 39 *GhSWEET* genes. (**B**) *Fst* values of the SNP loci in 39 *GhSWEET* genes. The SNPs with a *Fst* value greater than 0.45 were considered to be loci under selection during domestication.

The increase in the available re-sequencing data for wild and cultivated cotton species has enabled the analysis of the changes in *GhSWEET* genes that occurred during cotton domestication. In this study, the single nucleotide polymorphisms (SNPs) in all *GhSWEET* genes were obtained for the wild and domesticated species using re-sequencing data from 31 wild and 321 cultivated cotton varieties (Table S5). A total of 16 genes lacked SNPs in the wild and domesticated cotton lines. In the wild species, 37 *GhSWEET* genes had at least one SNP, and the SNPs of 26 *GhSWEET* genes were detected in exons. In the domesticated species, 37 *GhSWEET* genes included SNPs, and the SNPs of 27 genes were present in exons. Additionally, the Dt subgenome comprised more *SWEET* genes with SNPs in exons than the At subgenome. Further analyses revealed that these genes with SNPs were distributed in the four clades of the phylogenetic tree. Specifically, Clade III members had 55 SNPs, i.e., more SNPs than the members of the other clades. Additionally, *GhSWEET42* had 15 SNPs, i.e., more than any other gene. A subsequent calculation of the SNP density of all *GhSWEET* genes revealed that the SNP density of 10 *SWEET* genes (including eight At subgenome genes) in domesticated cotton was lower than in wild cotton (Figure 8). Moreover, *GhSWEET19* and *GhSWEET37* in domesticated upland cotton had no SNPs. Furthermore, the *Fst* values of the SNP loci during domestication indicated that 65.2% of the loci (88 of 135 SNP loci) were not under selection during evolution (*Fst* < 0.15). However, nine SNPs in seven *G. hirsutum SWEET* genes (*GhSWEET14*, *GhSWEET16*, *GhSWEET32*, *GhSWEET37*, *GhSWEET42*, *GhSWEET45*, and *GhSWEET54*) satisfied the *Fst* value cutoff of 0.45. These results indicated that domestication might have a critical effect on cotton *SWEET* genes, perhaps because of their importance in cotton development and response to various stresses.

## 3. Discussion

The SWEET proteins have fundamental roles affecting sugar transport in plants, animals, and microorganisms. Genome-wide studies of the *SWEET* sugar transporter genes have been conducted for many plants [14,21]. However, a genome-wide characterization and functional analysis of cotton *SWEET* genes is still lacking. Additionally, there is increasing evidence that cotton SWEET proteins are crucial for stress responses and fiber development [30,31,38]. Thus, the comprehensive analysis of the identified *G. hirsutum SWEET* genes described herein may provide valuable information to elucidate the role of the corresponding gene family in cotton species.

### 3.1. Expansion of the Gossypium SWEET Gene Family

*G. hirsutum* is an allotetraploid species that resulted from the hybridization between an A-genome species resembling *G. arboreum* and a D-genome species resembling *G. raimondii* [29,39]. In the present study, 31, 30, and 55 *SWEET* genes were identified in the *G. raimondii*, *G. arboreum*, and *G. hirsutum* genomes, respectively. The fact that the *SWEET* genes are more abundant in cotton species than in *A. thaliana* (17), rice (21), grapevine (17), tomato (29), and cucumber (17) suggests that the cotton *SWEET* gene family expanded during evolution. Previous studies concluded that a *Gossypium*-specific whole-genome duplication (WGD) event occurred in the diploid cotton species *G. raimondii* and *G. arboreum* [33,40,41]. Therefore, the expansion of the *SWEET* gene family in *G. raimondii* and *G. arboreum* may have been due to an ancient WGD, with a subsequent polyploidization event leading to duplicate copies of genes in the *G. hirsutum* genome. Meanwhile, some *SWEET* gene clusters were detected in the *G. hirsutum* At and Dt subgenomes. Tandem duplication events have been important for the expansion of many gene families [42,43,44]. Therefore, we speculated that tandem duplications might have amplified some *SWEET* genes, leading to the emergence of *SWEET* gene clusters.

The *SWEET* genes are divided into four clades based on their phylogenetic relationships, with cotton presenting more Clade III and IV genes than other analyzed plant species. For example, the *A. thaliana*, rice, grapevine, tomato and cucumber genomes contain two [12], one [13], four [19], two [20] and three [21] Clade IV *SWEET* genes, respectively, i.e., fewer than the 7, 6, and 12 Clade IV *SWEET* genes present in the *G. raimondii*, *G. arboreum*, and *G. hirsutum* genomes, respectively. These differences suggested that Clade III and IV genes might be vital for the adaptation of cotton species to environmental conditions.

### 3.2. Expression Patterns of SWEET Gene Family

The spatiotemporal expression profiles of the *SWEET* genes have been reported for some species, including *A. thaliana*, rice, soybean, and cucumber. Most soybean *SWEET* genes are highly expressed in developing flowers and seeds [14]. Additionally, the eight tested *CsSWEET* genes from the four evolutionary clades were expressed at very high levels in male and female flowers [21]. In this study, we analyzed the *GhSWEET* transcript levels in seven tissues. According to our analyses of phylogenetic clusters (Figure 2A), similar exon-intron structures among most members of the same subfamily resulted in similar expression patterns in different tissues. More than half of the examined *SWEET* genes were predominantly expressed in the root, flower, ovule, and fiber, with relatively low expression levels in the stem and seed. Physiologically, the root, which absorbs nutrients from the soil, is the most fundamental organ with essential roles related to the maintenance of normal plant growth and development. Additionally, the flower, ovule, and fiber of cotton plants are associated with organogenesis, signal transduction pathways, and various metabolic processes. Our results implied that SWEET proteins exhibited diverse functions affecting cotton development and growth.

Previous studies revealed that *SWEET* genes not only influence plant growth, but they are also important for controlling plant responses to environmental stresses and phytohormones [15,20]. Thus, we investigated *G. hirsutum SWEET* gene expression patterns in response to different stresses (Figure 6). We observed that the expression levels of six *GhSWEET* genes were significantly upregulated by all four of the chosen treatments. A qRT-PCR analysis of five *SWEET* genes of them revealed that the expression levels of five of these *SWEET* genes were significantly up-regulated under heat and drought stress conditions (Figure 7). Specifically, two genes (*GhSWEET20* and *GhSWEET51*) and four genes (*GhSWEET5*, *GhSWEET20*, *GhSWEET49*, and *GhSWEET55*) were considerably increased within 1 h of heat treatment and drought treatment, respectively. Therefore, we deduced that these genes have important roles in cotton stress resistance. In the future, these genes could be useful targets for breeding new cotton varieties with enhanced stress tolerance. Additionally, several hormone-responsive regulatory elements were identified in *GhSWEET* promoters (Table S4). This observation indicated that some *SWEET* genes might be expressed as part of phytohormone signaling pathways.

### 3.3. Functional Divergence of the Gossypium hirsutum SWEET Gene Family

Duplicated genes generally face one of three fates: non-functionalization, sub-functionalization, or neo-functionalization [45,46]. In this study, almost half of the duplicated gene pairs exhibited no differences in expression. These genes may have retained some essential functions based on gene dosage during evolution [47,48]. However, some paralogous *SWEET* gene pairs were associated with tissue-dependent diverse functions during plant growth and development. We observed that *GhSWEET16* and *GhSWEET33* were highly expressed in the root, leaf, and fiber, whereas their paralogous counterparts, *GhSWEET12* and *GhSWEET37*, were silenced in these tissues (Figure 5). Thus, we speculated that the non-functionalization of one member of a duplicated gene pair might have been due to the maintenance of an appropriate gene dosage during cotton evolution. Additionally, one member of some duplicated gene pairs underwent neo-functionalization via WGD and polyploidy events. For example, *AtSWEET1* is crucial for the regulation of floral development in *A. thaliana* [12]. In this study, its cotton orthologs (*GhSWEET50* and *GhSWEET52*) influenced flowering, but were also abundantly expressed in the fiber, suggesting that these genes may contribute to cotton fiber development. In contrast, *GhSWEET44* was specifically expressed in the flower, while its paralogous counterpart, *GhSWEET48*, was highly expressed in the root and flower. The variability in the expression profiles of paralogs in *G. hirsutum* may have been caused by several complex regulatory activities following gene duplication events. 

Tandem duplications may have significantly affected the divergence of gene functions during evolution [49,50]. We examined the expression patterns of 14 tandem duplicated genes from seven clusters (Figure 5). As a result, all tandem duplicated pairs were differentially expressed. For example, *GhSWEET32* was characteristically expressed in the leaf, while its tandem duplicated gene, *GhSWEET33,* was predominately expressed in the root, leaf and fiber. *GhSWEET46* was specifically expressed in the stem, while its tandem duplicated gene, *GhSWEET47*, was abundantly expressed in developing flowers. This implied that tandem duplicated genes in the tetraploid cotton species might have exhibited functional redundancies and evolved new functions.

To date, the functions of some *A. thaliana* and rice SWEET proteins have been determined [12,13]. However, there is a relative lack of information regarding the functions of cotton SWEET proteins. In the present study, we provide evolutionary and functional insights into the roles of cotton *SWEET* gene family which will help future functional studies of the *SWEET* genes during cotton development and stress responses.

## 4. Materials and Methods

### 4.1. Identification and Characterization of G. hirsutum SWEET Genes

The *G. hirsutum* genome sequence and annotation files [39] were downloaded from the CottonGen website (available online: https://www.cottongen.org). Additionally, the related sequences of published *A. thaliana* and rice *SWEET* genes [13,14] were obtained from the TAIR 10 database (available online: http://www.arabidopsis.org) and the Rice Genome Database (http://www.ricedata.cn/gene/), respectively and their encoded protein sequences were used as queries in BLASTP searches of the *G. hirsutum* genome data. Next, putative SWEET proteins were filtered on the basis of the presence of a conserved MtN3_saliva domain using Pfam database analyses (*E*-value cut-off of 1.0; http://pfam.xfam.org/search#searchBatchBlock). The candidate *SWEET* genes were subsequently aligned. Meanwhile, inconsistencies in the sequence alignment were resolved and absent sequences were predicted using FGENESH software [51] and cloned to determine the complete sequences. The *G. raimondii* [33] and *G. arboreum* [40] *SWEET* genes were identified using similar methods.

The *SWEET* genes were named according to their chromosomal locations in *G. hirsutum*. Other related biological details, including the number of amino acids, molecular weight (MW), and isoelectric point (pI), were calculated using the DNAMAN (version 9.0) software (Lynnon Biosoft, Quebec City, QC, Canada).

### 4.2. Phylogenetic Analysis

Phylogenetic trees were constructed using full-length SWEET protein sequences and the neighbor-joining method of the MEGA (version 7.0) (Tokyo Metropolitan University, Tokyo, Japan) program with the pairwise gap deletion option [52]. The bootstrap analysis was conducted with 1000 replicates.

### 4.3. Gene Structural Features and Chromosomal Localization

Exon-intron structures were analyzed using the GSDS server (available online: http://gsds.cbi.pku.edu.cn/) [53]. Meanwhile, the physical positions of all cotton *SWEET* genes on chromosomes were determined according to the genome annotation files downloaded from the CottonGen website (available online: https://www.cottongen.org). The *SWEET* genes were mapped using MapInspect software [54] or Circos (version 0.69) software [55]. Tandem duplications were analyzed on the basis of the same or neighboring chromosomal regions [56]. Homologous genes including paralogous and orthologous genes were identified according to phylogenetic trees and sequence alignments [57,58].

### 4.4. RNA-Sequencing Analysis

To analyze the *G. hirsutum SWEET* gene expression profiles, the transcriptome data for various *G. hirsutum* TM-1 tissues were downloaded from the NCBI Sequence Read Archive (accession number PRJNA248163; available online: https://www.ncbi.nlm.nih.gov/bioproject/PRJNA248163/). The gene expression levels were calculated on the basis of the normalized fragments per kilobase of transcript per million mapped fragments (FPKM). Hierarchical clustering was depicted using the Genesis (version 1.7.7) program [59].

### 4.5. Stress Treatments

Cotton seeds (TM-1) were sown in plastic pots filled with sand for about 10 days at 28 °C. The seedlings were transferred to a liquid culture medium under a 16h light-8h dark photoperiod until the third true leaf appeared. The cotton seedlings were treated with 20% PEG 6000 or heated at 38 °C. The leaves harvested at 0, 1, 3, 6, and 12 h, were immediately frozen in liquid nitrogen and stored at −80 °C for subsequent total RNA extraction.

### 4.6. RNA Isolation and Quantitative Real-Time Polymerase Chain Reaction Analysis

Total RNA was extracted from *G. hirsutum* leaves using the TRIzol reagent (Tiangen, Beijing, China). The quality and quantity of the purified RNA were assessed by 1.5% gel electrophoresis and with a NanoDrop 2000 spectrophotometer. For each sample, 1 μg RNA was reverse transcribed to cDNA using the PrimeScript™ RT Reagent Kit with gDNA Eraser (TaKaRa, Tokyo, Japan). Gene-specific primers were designed using the Primer-BLAST program (available online: https://www.ncbi.nlm.nih.gov/tools/primer-blast/index.cgi?LINK_LOC=BlastHome) (Table S1). The *G. hirsutum His3* housekeeping gene was used as an internal control. A quantitative real-time polymerase chain reaction (qRT-PCR) was conducted with a Lightcycler480 96 system (Roche, Mannheim, Germany) in a 20 μL reaction mixture containing SYBR Premix Ex Taq (2×) (TaKaRa). The qRT-PCR was completed with three biological replicates, each comprising three technical replicates. The PCR conditions were as follows: 95 °C for 30 s; 40 cycles of 95 °C for 5 s, 60 °C for 1 min, and 72 °C for 10 s; 50 °C for 30 s. The relative gene expression levels were calculated based on the 2^−ΔΔ*C*T^ method [60]. Finally, the gene expression patterns were analyzed using the Origin 8 software (OriginLab Corporation, Northampton, MA, USA).

### 4.7. Analysis of Genetic Variations and Artificial Selection of G. hirsutum SWEET Genes 

The whole genome re-sequencing data for 31 wild and 321 cultivated cotton lines were downloaded from the NCBI Sequence Read Archive (accession number SRP080913) [61]. Single nucleotide polymorphisms (SNPs) in *SWEET* genes were detected, and *Fst* values were calculated using Genepop (version 4.0) [62]. The SNP loci with a *Fst* value > 0.45 were identified as putative sites under selection during domestication.

## 5. Conclusions

In this study, we comprehensively analyzed *G. hirsutum SWEET* genes regard to their classification, phylogenetic relationships, structure, *cis*-acting regulatory elements, chromosomal localization, genetic variations, and expression patterns in diverse tissues and in response to various stresses. Our findings revealed that the *G. hirsutum SWEET* gene family expanded during evolution, perhaps because of a WGD, some tandem duplications, and a polyploidy event. The *GhSWEET* genes have significant roles related to cotton development and stress responses. The data presented herein provide a foundation for future functional studies of the most interesting cotton *SWEET* genes. Our findings may also be relevant for breeding new cotton varieties with enhanced stress tolerance and for accelerating research regarding cotton development.

## Figures and Tables

**Table 1:** Characteristics of the *G. hirsutum SWEET* genes.

Gene Name	Locus ID	Protein Length (aa)	MW (Da)	pI	Chromosome	Strand	Location
*GhSWEET1* ^a^	Gh_A01G0160	308	34,313.8	6.39	A01	Plus	1,535,209—1,538,355
*GhSWEET2* ^a^	Gh_D01G0202	311	34,634.0	5.58	D01	Plus	1,717,547—1,720,877
*GhSWEET3*	Gh_A02G0694	252	27,923.1	10.03	A02	Plus	11,719,940—11,721,641
*GhSWEET4*	Gh_A02G0950	295	33,137.6	6.99	A02	Minus	39,459,757—39,462,756
*GhSWEET5* ^a^	Gh_D02G0542	237	25,846.9	8.41	D02	Minus	7,239,140—7,241,801
*GhSWEET6*	Gh_D02G0740	252	27,953.1	10.03	D02	Plus	11,060,974—11,062,676
*GhSWEET7*	Gh_D02G1767	283	31,651.9	7.84	D02	Minus	60,142,543—60,144,477
*GhSWEET8* ^a^	Gh_A03G0461	315	36,712.1	9.97	A03	Plus	10,189,210—10,190,467
*GhSWEET9*	Gh_D03G0812	255	28,298.8	9.88	D03	Plus	27,825,277—27,828,205
*GhSWEET10*	Gh_D03G1078	306	34,864.5	9.09	D03	Minus	35,954,015—35,955,277
*GhSWEET11*	Gh_A04G0861	289	32,503.8	7.90	A04	Minus	55,950,153—55,951,883
*GhSWEET12*	Gh_D04G0509	257	28,434.0	9.08	D04	Plus	8,687,271—8,689,780
*GhSWEET13*	Gh_D04G0510	237	26,541.9	9.22	D04	Plus	8,700,584—8,702,611
*GhSWEET14*	Gh_D04G1360	289	32,430.8	7.90	D04	Minus	44,257,813—44,259,535
*GhSWEET15*	Gh_A05G3127	237	26,556.0	9.22	A05	Minus	80,724,645—80,726,627
*GhSWEET16* ^a^	Gh_A05G3128	257	28,415.9	9.41	A05	Minus	80,737,385—80,739,919
*GhSWEET17* ^a^	Gh_D05G1448	230	26,064.4	7.18	D05	Minus	12,908,186—12,909,403
*GhSWEET18* ^a^	Gh_A07G0421	276	30,996.2	9.64	A07	Plus	5,396,344—5,398,990
*GhSWEET19*	Gh_A07G0422	280	31,849.3	9.74	A07	Plus	5,417,794—5,419,168
*GhSWEET20*	Gh_A07G0423	277	31,350.6	9.61	A07	Plus	5,441,891—5,443,770
*GhSWEET21* ^a^	Gh_A07G0535	235	26,705.5	9.96	A07	Minus	7,195,579—7,196,830
*GhSWEET22*	Gh_D07G0486	274	30,709.0	9.63	D07	Plus	5,386,349—5,388,888
*GhSWEET23* ^a^	Gh_D07G0487	280	31,876.4	9.74	D07	Plus	5,393,200—5,394,555
*GhSWEET24* ^a^	Gh_D07G0604	235	26,719.5	9.96	D07	Minus	6,953,608—6,954,845
*GhSWEET25*	Gh_A08G0663	275	30,950.1	9.18	A08	Minus	13,836,649—13,837,903
*GhSWEET26* ^a^	Gh_D08G1194	258	22,445.5	9.80	D08	Plus	38,403,775—38,405,327
*GhSWEET27* ^a^	Gh_A09G1524	250	13,186.5	9.50	A09	Minus	68,504,026—68,505,455
*GhSWEET28*	Gh_A10G1468	229	24,973.0	7.40	A10	Minus	79,914,294—79,915,964
*GhSWEET29* ^a^	Gh_D10G0303	230	20,367.1	9.64	D10	Minus	2,608,669—2,610,376
*GhSWEET30*	Gh_D10G1709	229	24,987.0	7.40	D10	Minus	47,620,123—47,621,535
*GhSWEET31*	Gh_A11G0347	298	33,545.7	8.12	A11	Minus	3,182,270—3,183,655
*GhSWEET32* ^a^	Gh_A11G2442	238	26,970.4	7.89	A11	Minus	82,756,807—82,758,639
*GhSWEET33*	Gh_A11G2446	300	32,893.8	9.72	A11	Minus	82,867,355—82,869,323
*GhSWEET34*	Gh_A11G2655	251	28,283.5	9.25	A11	Plus	88,688,141—88,689,610
*GhSWEET35*	Gh_D11G0404	298	33,559.8	8.34	D11	Minus	3,381,068—3,382,434
*GhSWEET36* ^a^	Gh_D11G2760	234	26,215.2	8.15	D11	Minus	57,260,671—57,262,412
*GhSWEET37* ^a^	Gh_D11G2763	301	33,196.0	8.79	D11	Minus	57,340,382—57,342,337
*GhSWEET38*	Gh_D11G2975	234	26,205.7	9.32	D11	Plus	60,589,364—60,591,634
*GhSWEET39* ^a^	Gh_A12G1747	295	33,121.3	7.09	A12	Minus	79,539,983—79,541,579
*GhSWEET40*	Gh_A12G2152	254	28,192.6	10.07	A12	Minus	84,324,046—84,326,052
*GhSWEET41* ^a^	Gh_D12G1898	295	33,145.2	6.39	D12	Minus	51,822,209—51,825,243
*GhSWEET42*	Gh_D12G2328	254	28,177.6	9.98	D12	Minus	56,415,133—56,417,086
*GhSWEET43*	Gh_A13G0907	249	26,768.6	8.59	A13	Minus	47,687,824—47,689,812
*GhSWEET44* ^a^	Gh_A13G1434	269	30,513.0	8.89	A13	Plus	72,034,747—72,035,999
*GhSWEET45*	Gh_A13G1540	245	27,174.2	8.60	A13	Minus	73,975,893—73,977,109
*GhSWEET46* ^a^	Gh_D13G1146	249	27,432.3	7.43	D13	Minus	34,001,023—34,002,217
*GhSWEET47*	Gh_D13G1148	248	26,555.3	9.14	D13	Minus	34,107,380—34,109,414
*GhSWEET48* ^a^	Gh_D13G1763	269	30,478.9	8.89	D13	Minus	52,413,794—52,414,799
*GhSWEET49*	Gh_D13G1875	245	27,108.2	8.60	D13	Minus	53,858,190—53,859,414
*GhSWEET50*	Gh_A02G1806	250	27,450.6	9.85	scaffold476_A02	Plus	194,323—196,496
*GhSWEET51*	Gh_A11G3285	235	26,286.6	8.85	scaffold3047_A11	Minus	190,443—192,707
*GhSWEET52*	Gh_D03G1717	250	27,574.7	9.37	scaffold3920_D03	Plus	575,817—577,994
*GhSWEET53*	Gh_D08G2730	275	30,879.1	9.18	scaffold4310_D08	Plus	24,770—26,035
*GhSWEET54* ^a^	Gh_D12G2692	252	16,414.6	8.47	scaffold4580_D12	Plus	11,296—12,765
*GhSWEET55*	Gh_Sca013374G01	273	30,600.0	9.85	scaffold13374	Plus	471—1,821

^a^ The coding sequences of genes are manually re-annotated. MW: Molecular weight; pI: Isoelectric point.

## Supplementary Materials

Supplementary materials can be found at http://www.mdpi.com/1422-0067/19/3/769/s1. Table S1. Sequences of qRT-PCR primers of the selected *GhSWEET* genes and the *GhHis3* internal reference gene. Table S2. Coding sequences of *G. hirsutum SWEET* genes. Table S3. Details regarding *G. raimondii* and *G. arboreum SWEET* genes. Table S4. Analysis of *cis*-acting regulatory elements in the promoters of putative *GhSWEET* genes responsive to environmental stresses and phytohormones. Table S5. *Fst* values of the SNPs in *GhSWEET* genes between wild and cultivated upland cotton lines. Figure S1. Distribution of orthologous pairs of *SWEET* genes between the *G. hirsutum* Dt subgenome and *G. raimondii* D genome (A) and between the *G. hirsutum* At subgenome and *G. arboreum* A genome (B). Different colored lines represent *SWEET* genes in four clades based on phylogenetic trees.

## Abbreviations

SWEET	Sugars will eventually be exported transporters
SUT	Sucrose transporter
ABA	Abscisic acid
SA	Salicylic acid
GA	Gibberellin
MeJA	Methyl jasmonate
BR	Brassinosteroid
MW	Molecular weight
pI	Isoelectric point
FPKM	Fragments per kilobase of transcript per million mapped fragments
qRT-PCR	Quantitative real-time polymerase chain reaction
SNP	Single nucleotide polymorphism
WGD	Whole-genome duplication
