# Supplementary material for: Evolution and Stress Responses of Gossypium hirsutum SWEET Genes
Source: Int J Mol Sci. 2018 Mar 8;19(3):769. doi: 10.3390/ijms19030769 (PMC5877630; doi:10.3390/ijms19030769)
Supplement: Supplementary file 1 [file ijms-19-00769-s001.zip › ijms-275578-Suppl/ijms-275578-Suppl.doc]

*Article*

**Evolution and Stress Responses of
*Gossypium hirsutum SWEET* Genes**

**Wei Li, Zhongying Ren, Zhenyu Wang, Kuan Sun, Xiaoyu Pei, Yangai Liu, Kunlun He,
Fei Zhang, Chengxiang Song, Xiaojian Zhou, Wensheng Zhang, Xiongfeng Ma
and Daigang Yang**

**Figure S1.** Distribution of orthologous pairs of *SWEET* genes between the *G. hirsutum* Dt subgenome and *G. raimondii* D genome (**A**) and between the *G. hirsutum* At subgenome and *G. arboreum* A genome (**B**). Different colored lines represent *SWEET* genes in four clades based on phylogenetic trees.

**Table S1.** Sequences of qRT-PCR primers of the selected *GhSWEET* genes and the *GhHis3* internal reference gene.

| Gene Name | Forward Primer (5′–3′) | Reverse Primer (5′–3′) |
| --- | --- | --- |
| *GhSWEET5* | TCTACTGGGAACAGCACAACT | GGGGCTCCTTTTGTTCCATT |
| *GhSWEET20* | CCCAAAGAAAATGGTGGTGGAAG | TCTTTGTCTTTCGTTTGAGGAGC |
| *GhSWEET49* | TAATGGCTTTGGTGCTGTCCT | TTATGGCTCTCGCTCTGGGA |
| *GhSWEET51* | GGCTAGTGTTCTTTGGTTGGC | AATCCCATTTGCTTGGTTCTTCTT |
| *GhSWEET55* | TACCATCAACACTTTCTGCGTTT | AAATCAGGAGACGAAGGGAAGG |
| *GhHis3* | TCAAGACTGATTTGCGTTTCCA | GCGCAAAGGTTGGTGTCTTC |

© 2018 by the authors. Submitted for possible open access publication under the
terms and conditions of the Creative Commons Attribution (CC BY) license (http://creativecommons.org/licenses/by/4.0/).
